# Do Defective Immune System-Mediated Myelination Processes Increase Postpartum Psychosis Risk?

**DOI:** 10.1016/j.molmed.2018.09.002

**Published:** 2018-11

**Authors:** Paola Dazzan, Montserrat Fusté, William Davies

**Affiliations:** 1 Department of Psychosis Studies, Institute of Psychiatry, Psychology and Neuroscience, Kings College London, London, UK; 2 National Institute for Health Research (NIHR) Mental Health Biomedical Research Centre at South London and Maudsley NHS Foundation Trust and King’s College London, London, UK; 3 Medical Research Council Centre for Neuropsychiatric Genetics and Genomics and Neuroscience and Mental Health Research Institute, Schools of Medicine and Psychology, Cardiff University, Cardiff, UK

**Keywords:** bipolar disorder, CCN3, mouse model, multiple sclerosis, oligodendrocyte, regulatory T cell

## Abstract

Postpartum (or puerperal) psychosis (PP) is a rare, severe psychiatric disorder that affects women shortly after childbirth; risk is particularly high in individuals with a history of bipolar disorder or PP, but the underlying pathophysiology remains poorly understood. Emerging evidence suggests that immune system (dys)function plays an important role in disorder onset. On the basis of new findings from clinical and animal model studies, we hypothesise that the abundance and/or activity of regulatory T cells, and the efficacy of consequent (re)myelination processes in the brain mediated by CCN proteins, is perturbed in PP; this pathway may be modulated by risk and protective/treatment factors for the disorder, and identifying abnormalities within it could signpost novel predictive biomarkers and therapeutic targets.

## Highlights

Postpartum psychosis (PP) is a rare, severe psychiatric disorder affecting some women shortly after childbirth.

While the risk factors for PP are relatively well understood, our knowledge of the underlying pathophysiology is extremely poor; however, there is some evidence that immune system dysregulation may play a role.

A recent study has shown that regulatory T cells (Tregs) may promote remyelination via the CCN family of proteins.

Here, we argue, on the basis of emerging immunological, animal model, and neuroimaging findings, that PP risk may be elevated due to abnormalities in the Treg–CCN–(re)myelination axis and that risk and protective/treatment factors for the disorder may influence this axis.

## Symptoms and Pathophysiology of Postpartum Psychosis

Postpartum psychosis (PP) is a severe psychiatric disorder affecting approximately one or two of every 1000 women shortly after childbirth (most often within 6 weeks); the main symptoms include hallucinations, delusions, cognitive disorganisation, and mood abnormalities [1]. PP risk is highly predictable, with a risk recently reported to be ∼35% for women with a history of either bipolar affective disorder or previous PP [2]. Although PP occurs concomitantly with the biological sequelae of childbirth, its neurobiological basis remains poorly understood.

The most intensively studied biological changes in PP have been those that occur in maternal reproductive hormones shortly after childbirth; however, no differences in absolute levels of oestrogen or progesterone have been identified in women who develop an episode of PP 3, 4. A genetic liability to PP has also been suggested: a linkage study in women with bipolar affective PP implicated chromosomal regions 16p13 and 8q24 (regions previously associated with bipolar disorder vulnerability) [5], while genetic association studies have provided suggestive, albeit inconclusive, evidence of risk variants within candidate genes involved in serotonergic, hormone-dependent, or stress-response pathways 6, 7, 8.

Key obstacles in identifying biological risk and protective factors for PP include low disorder prevalence, high between-patient symptom heterogeneity, lack of accessibility to patient brain tissue, and a historical lack of amenable model systems [9]. However, understanding the pathophysiology of PP is crucial for identifying both novel therapeutic targets (for more efficacious drugs with fewer adverse effects) and predictive biomarkers (for identification of the most ‘at risk’ women before, or during, pregnancy, thereby facilitating earlier, more individualised clinical interventions).

Recent evidence has highlighted a potentially important role for the immune system in the onset of PP: women with the disorder have higher rates of autoimmune thyroid dysfunction and pre-eclampsia (considered a disease of immunological maternal-fetal incompatibility) 1, 10, and some cases of PP involve an autoimmune response against the *N*-methyl-d-aspartic acid receptor [11]. The link between PP pathophysiology and the immune system may not be surprising, given that immune system dysregulation, characterised by elevated levels of serum proinflammatory cytokines such as interleukin (IL)-6, IL-1, and tumour necrosis factor-α, and alterations in the expression of associated genes now features prominently in pathophysiological models of depression (including in the postpartum period) 12, 13, bipolar affective disorder [14], and psychosis 15, 16.

## Immune System Dysfunction and PP

PP occurs at a time of naturally heightened immune responsiveness. In pregnancy, a predominant anti-inflammatory T helper (Th)2-type immunity (whereby Th2 cell activity, including cytokine expression, is favoured over that from Th1 cells), has been historically considered responsible for maternal tolerance toward fetal alloantigens to protect the foetus from maternal Th1 cell-mediated immunity and subsequent attack [17]. However, more recent models of immunity function in pregnancy also implicate other T cell types, including **regulatory T cells** (Tregs; see Glossary), cytotoxic natural killer (NK) cells, and Th17 cells 18, 19. Treg levels typically increase in late pregnancy and peak early in the postpartum period, to be followed by later depletion [19]. Not only may Treg cells be important for regulating immunity and mediating self-tolerance in pregnancy but also for moderating the course of autoimmune disorders during this period. For example, the proliferation of Tregs that occurs during pregnancy is thought to mitigate acute exacerbations of multiple sclerosis (MS), an immune system-mediated demyelinating disease [20].

Two recent studies have reported that women with PP have lower levels of Th1, Th17, and Tregs [21] and a lower proportion of naïve Tregs (but a higher proportion of memory Tregs) [22] than healthy postpartum women. These findings may reflect the disrupted function of one or more subpopulations of Tregs in this clinical group, although the mechanisms underlying group differences in T cell proportions, and their precise functional relevance, are currently unclear. In addition, women with PP have been reported to exhibit lower numbers of NK cells [21] but increased regulatory NK cells [22] that counterbalance the cytotoxic NK response and are suggestive of a possible defect in the immune restoration normally present in the postpartum period. Furthermore, women with PP show a reduction in numbers of dendritic cells [22]; these cells are known to secrete higher levels of IL-12 and IL-6 in MS by inducing proinflammatory Th1 and Th17 cells, and their reduced numbers in women with PP could theoretically result in the persistence of immunosuppressive cells in the postpartum period.

Finally, monocyte and macrophage levels have also been found to be significantly higher in women with PP than in those without PP. Although Kumar and colleagues [22] found no difference in total monocyte numbers between women with PP and healthy postpartum women, their study did find lower levels of the non-classical monocyte subtype, which display inflammatory characteristics upon activation, in the former group. The parallel findings of higher blood levels of chemokine CCL2 (produced by monocytes during tissue infiltration) in women with PP, and of a robustly upregulated immune system-related gene expression profile in this clinical group, strongly support the relevance of monocyte alterations in this disorder [21].

Collectively, the above-mentioned data support a model for PP whereby an imbalance between proinflammatory and immunosuppressive cells of the immune system in the postpartum period may be strongly associated, possibly causally, with the development of the disorder. It remains to be established whether, and how, peripheral immune activation might affect the maternal brain processes that underlie PP onset. However, data relevant to the pathophysiology of MS and other psychiatric conditions, discussed in the subsequent sections, suggest that an influence on myelination processes is possible.

## Myelination Processes in the Perinatal Period

While gray and white matter abnormalities have been consistently reported in psychoses not related to the puerperium [23], until very recently, brain structure had never been investigated in PP. To date, only one study (from our group) has systematically evaluated gray matter structure using magnetic resonance imaging in women at risk of PP [24]. In brief, this study found that ‘at risk’ women who developed PP had smaller volumes of the anterior cingulate, superior temporal, and parahippocampal gyri compared to women at risk who did not develop PP, a pattern of alterations resembling that often found in patients with psychoses not related to the puerperium. Using a different imaging sequence, we also showed in this sample that women at risk of PP have reduced myelin content in the temporal lobes and sublobar areas relative to healthy women [25]. A case report in which PP was associated with white matter pathology in the corpus callosum [26] adds further weight to a possible link between PP risk and abnormal myelination processes.

Studying white matter structure using sufficiently sensitive methods may be particularly informative in the context of PP, given evidence that maternal brain structure and associated physiology change substantially during pregnancy and into the postpartum period 27, 28 and that there are notable sex differences in white matter structure, both in healthy individuals and in those affected by psychosis 29, 30. Of relevance to our proposed model, pregnancy is a time of considerable change in women with MS and in animal models of the condition, whereby symptom severity and the number/size of active white matter lesions are reduced [31]; this may be explained by the hormonal changes that physiologically occur in pregnancy (notably changes in prolactin levels), which can stimulate the maternal central nervous system (CNS) to generate new myelinating **oligodendrocytes** and facilitate partial remyelination, even in the presence of immune activation [32]. We propose that the (re)myelination processes that occur during pregnancy and the postpartum period in MS patients (and possibly also in healthy individuals) are compromised in women at risk of PP; this may explain why bipolar disorder symptoms do not improve significantly during pregnancy 33, 34.

## A New Hypothesis Reconciling Immune System Dysfunction and Myelination Abnormalities in PP

The recent publication of a study examining the cellular and molecular underpinnings of MS prompted us to consider previously unexplored mechanisms that may contribute to PP vulnerability. This new study elegantly demonstrated that Tregs can promote oligodendrocyte differentiation and (re)myelination within the CNS independently of inflammatory processes via secretion of the protein CCN3 (also known as nephroblastoma-overexpressed protein, or NOV) [35]. This finding was of particular interest to us given that the **CCN protein family** member CCN3 had been suggested as a candidate mediator of PP risk by our work in a novel pharmacological mouse model [36]. In our model, *Ccn3* expression was upregulated in whole brain tissue from new mouse mothers acutely administered an inhibitor of steroid sulfatase (to mimic the putative PP risk factor maternal steroid sulfatase deficiency [37]); these mice exhibited abnormal postpartum anxiety-related and startle behaviours of possible relevance to PP. The behavioural abnormalities and the *Ccn3* overexpression in the mouse model could be attenuated through administration of the antipsychotic drug ziprasidone, supporting its face and predictive validity. The finding that manipulation of placental gene activity in mice gives rise to altered maternal behaviours and increased brain (hippocampal) *Ccn3* expression [38] further supports the notion of CCN3 as a mediator of postpartum psychopathology 9, 36.

Our pharmacological model also revealed increased brain gene expression for putative CCN protein interactors, including the pro-depressant CCN2(CTGF) [39], which may heterodimerise with CCN3 and exert antagonistic effects [40], and the PP-relevant CCL2 protein 36, 41. CCN2 blocks oligodendrocyte differentiation to reduce myelination levels 42, 43 while CCL2 (monocyte chemoattractant protein-1) can influence blood–brain barrier permeability [44] and mobilisation of oligodendrocyte progenitor cells [45].

The CCN3 protein is present in the extracellular matrix, the cytoplasm, and the nucleus, and is involved in many diverse cellular processes including cell adhesion, migration, proliferation, differentiation, and survival [46]; it possesses multiple functional characteristics that support its candidacy as a mediator of PP risk, and its associated gene is located within the candidate PP genetic risk locus at 8q24 9, 36. In adult human brain, *CCN* gene family members, including *CCN3*, are highly expressed throughout the cortex and the limbic system [46], and their expression in these, and other tissues, may be modulated by several PP-relevant molecules such as oestrogen, serotonin, cytokines, psychotomimetics, and the mood-stabilising drug lithium 9, 36.

The intriguing findings described above, together with emerging knowledge of immune system and brain structural alterations in PP, have allowed us to generate a novel and testable evidence-based hypothesis regarding PP risk that reconciles these lines of evidence. Generally, we propose that women at risk of PP are more likely than healthy individuals to display abnormalities in the immune system–CCN family (and interactors)–(re)myelination axis and that disruption of this axis is a core pathophysiological feature of PP. Specifically, we propose that a perturbation in Treg abundance/activity in women at risk of PP may be associated with altered expression of the CCN3 protein in the brain and subsequently with reduced levels of myelination in cortical and limbic brain regions exhibiting the highest *CCN3* expression and/or with the greatest accessibility to Treg influx.

## Modulation of the Treg–CCN3–Myelin Axis by Risk and Protective/Treatment Factors for PP

If dysfunction of the aforementioned axis contributes significantly to PP pathophysiology, we might expect key risk and protective factors associated with the condition to impact upon it. Patients with bipolar disorder show consistent evidence for reduced numbers of circulating Tregs 47, 48, and this is correlated with white matter integrity across much of the brain [49]; the molecular mechanisms underlying this correlation are, as yet, undetermined. Similarly, patients with autoimmune thyroid diseases have been reported to exhibit reduced Treg numbers [50] or impaired Treg cell function [51]. There is convincing evidence that the thyroid system can affect myelination and neural connectivity processes [52], and rodent work has demonstrated that CCN expression is regulated by the thyroid hormone triiodothyronine in brain cortex [53]. Pre-eclampsia is also associated with low levels of circulating Tregs in some cases [54], with long-term white matter changes (notably in the temporal lobe, an area involved in the pathophysiology of psychosis 55, 56), and with perturbed expression of CCN family members (including CCN3) in placental tissue and serum [57]. Moreover, CCN3 has repeatedly been implicated as a risk factor for hypertension [58], a core feature of pre-eclampsia.

Interestingly, systemic kynurenine levels (associated with effects on Treg activity [59]) have been reported to be lower in women with PP than in healthy controls [60], and reduced kynurenine levels in patients with bipolar disorder have been associated with impaired white matter integrity [61]. Furthermore, the fact that remyelination efficiency decreases with age [62] may feasibly explain the association between PP risk and older maternal age [63].

Mood-stabilising drugs such as lithium have been reported to enhance white matter integrity in adults with bipolar disorder 64, 65, most likely through stimulating (re)myelination-promoting molecular cascades in oligodendrocytes [66]. The effects of antipsychotic administration on white matter integrity appear inconsistent and are more poorly defined, but there is some evidence for enhanced myelination via effects on oligodendrocytes 67, 68. Finally, rates of PP are higher in non-smokers than in smokers with bipolar disorder [69]; this is interesting since smoking boosts numbers and/or function of Tregs in the large airways and lungs (and possibly other tissues) 70, 71 and reduces *Ccn3* expression in these tissues in a model system [72].

## Limitations of the Hypothesis

Some important limitations to this proposed new hypothesis should be considered. First, the immunological, neuroimaging, and animal model findings upon which the hypothesis is founded are provisional and have yet to be replicated and fully characterised. Moreover, to date, it has been challenging to examine links between subtly aberrant de- or remyelination processes and vulnerability to general psychoses due to technical and practical limitations; with the advent of techniques with improved reliability and sensitivity, it should be possible to address more effectively how the former might influence the latter [73]. Second, other studies in women with PP or using alternative experimental models have not yet provided evidence for our hypothesis, although they have not refuted it. For example, early results from genome-wide association studies in PP have not identified dysfunctional T cell, CCN-related, or myelination pathways; however, this ‘absence of evidence’ should be viewed in light of the fact that these genetic studies are currently significantly underpowered [9]. Similarly, genetic and gene expression data from another animal model for PP, the infanticidal sow pig, have not strongly highlighted these pathways, although altered brain expression of myelin basic protein (MBP), prolactin, and the antipsychotic-target dopamine receptor D2 was noted in this model [74]; this lack of convergence could be explained by the fact that gene expression was only examined in a single brain region (the hypothalamus) involved in maternal behaviour, but not yet strongly implicated in PP pathophysiology [9]. An association analysis in the infanticidal sow pig highlighted *PAX3* as a candidate gene [75]; PAX3 may act as a transcriptional activator or repressor at the *CCN3* [76] and *MBP* [77] genes, respectively.

Third, our hypothesis does not definitively identify the ‘postpartum trigger’. In a diathesis model, it is possible that dysregulation of the Treg–CCN3–myelination pathway confers trait vulnerability to PP and that the disorder then becomes manifest upon exposure to postpartum-specific factors, such as changes in hormone levels or environmental stressors. Alternatively, as studies aimed at understanding the molecular pathogenesis of bipolar disorder have not yet implicated CCN3 abnormalities, and as brain levels of CCN family proteins (and interactors) fluctuate considerably across mammalian pregnancy and the postpartum period [78], it is plausible that it is specific abnormalities in the expression/function of these proteins that confer PP risk.

Finally, it is unclear how Tregs may mediate myelination processes in the brain in women at risk, or with a history, of PP. Theoretically, this could be via secretion of factors such as CCN3 into the peripheral circulation and subsequent uptake into the brain. Alternatively, Tregs may percolate into the brain via a permeabilised blood–brain barrier (perhaps facilitated by increased CCL2 expression) and/or via the choroid plexus to affect function in the nearby limbic system [79]. While there is some evidence that Treg infiltration into the CNS may influence the presentation of MS (and possibly also bipolar disorder) 49, 79, to date there is no published information on the integrity of the blood–brain barrier in women with PP.

## Concluding Remarks

The precise mechanistic details by which Tregs might permeate the CNS, produce and secrete CCN3, and by which CCN3 may then influence oligodendrocyte-mediated (re)myelination processes remain to be clarified over the coming years. Identifying the effectors of these processes would allow a more focussed genetic, biochemical, and cellular investigation of the pathophysiology of PP. For example, genetic studies might focus upon specific candidate genes or pathways that are highlighted, negating the need for genome-wide multiple testing correction. A putative interactor with CCN3, discoidin domain receptor 1 (DDR1) [80], may warrant further analysis in PP: intriguingly, this gene/protein is highly expressed in human CNS myelin where it co-localises with MBP [81], is upregulated during oligodendrocyte-mediated (re)myelination [82], and is associated with psychotic illness [83]. In the shorter term, clinical studies in women at risk of, or with, PP might focus on evaluating the number, proportion, and subtype of circulating Tregs; the frequency of genetic variants within *CCN* family and *DDR1* genes; levels of CCN3 (and CCN2) gene/protein expression in peripheral tissues such as serum, cerebrospinal fluid, and urine during pregnancy and the postpartum period; and the degree/integrity of myelination in relevant limbic and cortical structures.

Our hypothesis may also be tested in more experimentally tractable systems, such as rodent models in which detailed measures of myelination can be obtained *in vivo* or postmortem using imaging and/or histological methods, respectively. In adult mice, Treg depletion in combination with exposure to environmental stressors results in phenotypes of relevance to PP (notably increased anxiety-related behaviours) together with decreased hippocampal serotonin levels [84]. We predict that Treg depletion and stressor exposure should elicit differential effects on maternal behaviour, *Ccn3* expression, hippocampal 5-hydroxytryptamine, and myelination levels in postpartum versus non-postpartum female mice, and versus control postpartum mice. Finally, gross manipulation of CCN3 levels *in vivo* (e.g., in knockout mice or in brain-overexpression models) or *in vitro* in mixed neuron–oligodendrocyte cell cultures may be associated with perturbed myelination and corresponding PP-relevant behavioural phenotypes. Likewise, in the pharmacological and genetic mouse models that display abnormal maternal behaviour and altered *Ccn3* expression 9, 36, 38, we might expect subtle changes in myelination markers.

We believe that the hypothesis described above provides a plausible and coherent central risk mechanism for PP, drawing together existing lines of evidence implicating risk and protective/treatment factors. The hypothesis makes specific and testable predictions across many clinical and experimental systems (see Outstanding Questions). Confirmation of abnormalities in the Treg–CCN3–myelin axis in PP might feasibly identify new avenues of treatment for PP (e.g., via modulation of Treg number/function [85] or CCN3 knockdown [86]), as well as novel predictive peripheral biomarkers (e.g., genetic polymorphisms, or perturbed peripheral CCN levels).

Finally, our idea suggests that not only studies in inflammatory and autoimmune conditions previously associated with PP (e.g., pre-eclampsia and thyroid disorders) but also studies investigating immune conditions associated with aberrant (re)myelination and neuropsychiatric phenotypes such as MS might be informative for understanding PP pathophysiology; as such, a greater degree of collaboration between clinicians and scientists working within these areas is warranted.

**Clinician’s Corner**

Postpartum psychosis (PP) is a rare, severe, psychiatric disorder affecting some women shortly after childbirth; risk is known to be substantially elevated in women with a previous history of bipolar disorder, schizoaffective disorder, or PP, but the underlying pathophysiology is extremely poorly understood. Moreover, approximately half of the time, PP occurs in women with no previous psychiatric history.

While the current treatments for PP are relatively efficacious, they do not target the cause of the disorder, they may be teratogenic if administered prophylactically during pregnancy, and they may have adverse side effects. There are currently no predictive markers available for PP; such markers would allow women at high risk to be identified early in pregnancy, thereby facilitating earlier, more individualised, and more effective clinical intervention.

Here, we hypothesise, based upon emerging immunological, animal model, and neuroimaging data, that PP risk may be mediated by dysfunction of the regulatory T cell–CCN protein family–(re)myelination axis, a pathway that is also potentially aberrant in multiple sclerosis (MS); hence, we suggest that increased collaboration between clinicians and basic scientists studying PP and MS is warranted.

The identification of abnormalities within the axis above in women at high risk of PP could ultimately allow the development of predictive biomarkers and more targeted treatment options with fewer side effects for this vulnerable group.

Outstanding Questions How do regulatory T cell (sub)populations differ between women who have experienced an episode of postpartum psychosis (or who are at high risk) and mothers who have not?

Does the sequence of *CCN2*, *CCN3*, and *DDR1* genes differ between women who have experienced an episode of postpartum psychosis (or who are at high risk) and mothers who have not?

Does the peripheral expression of *CCN2*, *CCN3*, and *DDR1* genes (and their associated proteins) differ between women who have experienced an episode of postpartum psychosis (or who are at high risk) and mothers who have not?

Do markers of (re)myelination efficacy differ between women who have experienced an episode of postpartum psychosis (or who are at high risk) and mothers who have not? If so, do these changes show any degree of regional specificity in the brain?

To what extent do between-group differences in regulatory T cell populations, the CCN system, and myelination markers correlate with one another?

Do animal and cellular models with experimental manipulations of regulatory T cell number/function and/or CCN expression exhibit anatomical, neurochemical, and behavioural features relevant to postpartum psychosis?

Might a better understanding of the molecular pathways mediating immune system-mediated remyelination pathways allow for better postpartum psychosis risk prediction (e.g., in genetic analyses)?

## Glossary

**CCN protein family**: the CCN family of proteins comprise six members, with the CCN abbreviation deriving from the first letters of the first three family members: CYR61 (CCN1), CTGF (CCN2), NOV (CCN3), WISP1 (CCN4), WISP2 (CCN5), and WISP3 (CCN6). CCN proteins are expressed both within the cell and in the extracellular matrix. They mediate a wide range of fundamental cellular processes including survival, proliferation, differentiation, senescence, adhesion, and migration.
**Oligodendrocytes**: oligodendrocytes are central nervous system cells, the primary function of which is to provide support and insulation to axons through forming a myelin sheath. In the adult brain, oligodendrocytes develop from oligodendrocyte progenitor cells.
**Regulatory T cells**: regulatory T cells are a subpopulation of the immune system’s T cell ensemble. They are immunosuppressive and act to ‘dampen’ the activity of effector T cells, thereby helping to maintain tolerance to self-antigens and prevent autoimmune conditions.
